# Attachment, behavior problems and interventions

**DOI:** 10.3389/frcha.2023.1156407

**Published:** 2023-04-21

**Authors:** Judy Hutchings, Margiad E. Williams, Patty Leijten

**Affiliations:** ^1^ Centre for Evidence Based Early Intervention (CEBEI), School of Human and Behavioural Sciences, Bangor University, Bangor, United Kingdom; ^2^ Research Institute of Child Development and Education, University of Amsterdam, Amsterdam, Netherlands

**Keywords:** childhood, attachment, behavior problems, parenting programs, behavioral analysis

## Abstract

This paper puts forward an explanation for the frequent co-occurrence of attachment and behavior problems in children and the implications of this for interventions; presents preliminary evidence that some behaviorally based parenting programs reduce child behavior problems through two separate, but mutually reinforcing, processes—improved attachment relationships and increased parental use of behavior management techniques; and suggests next steps for the field to improve outcomes for those children who, without interventions that addresses both relationship building and behavior management, are at risk of significant long-term difficulties.

## 1. Introduction

Significant attachment disorders/difficulties are frequently established early in children's lives (1) and behavioral challenges also emerge when children are quite young. Both have poor outcomes and, over time, often co-occur (2–6). This paper presents a brief behavioral analysis of attachment and reviews evidence that behavioral parenting programs, that include relationship building, conceptualized within a behavioral framework, improve child-parent attachment relationships whilst independently reducing child behavior problems.

## 2. Attachment

Children's very early experience of the care, provided by their primary carers, impacts their developing brain function in terms of their ability to regulate both physiology and behavior (1). Experience-dependent brain maturation contributes to an understanding of attachment supporting Bowlby's (7) view that attachment has a biological function that mediates processes in children's emotions, social cognition and behavior with studies demonstrating how children make the best use of the care that is offered and demonstrating relative deficits in effortful control or self-regulation among children with disorganized attachments (8–10).

Maternal attachment style, associated with internalizing and externalizing behavior in children, carries intergenerational risks associated with adverse maternal childhood experiences and current mental health problems (11). Caregiver sensitivity ensures secure infant attachment (12–14) with early caregiver sensitivity in low-income mothers of children aged 1 year predicting their self-regulation at ages 2 and 3 (15, 16). Furthermore these early attachment relationships patterns become increasingly hard to change over time.

Maternal depression significantly reduces maternal responsiveness, contributing to impaired bonding, early mother–baby interaction difficulties and long-term effects for children (17, 18) and there are risks particularly for children who are removed from their biological parent (1).

Attachment-based studies show both levels of cortisol that differ from those of securely attached children and effective changes in brain function and attachment behaviors following attachment based intervention [e.g., (1), in a their study with fostered toddlers]. However, much of this work focuses on children's very early years and seldom evaluates impacts on co-occurring behavioral problems. However, a recent meta-analysis of Video-feedback Intervention to Promote Positive Parenting and Sensitive Discipline, combining parental sensitive responsiveness and sensitive limit setting, (VIPP-SD) (19) showed substantial effects on parenting behavior and attitudes and child attachment security but no significant effects on child externalizing behavior. Furthermore despite “*an extensive literature linking maternal sensitivity, attachment and child outcomes …. at present the evidence base for directly affecting attachment via maternal sensitivity is equivocal”* (20).

## 3. Behavior problems

Early child behavior problems represent the largest referral category to child mental health services (21), have lifelong effects (2) and are extremely costly (21, 22). Longer term problems include poor health, school failure, delinquency, drug use, adult offending and violence towards partners and children (23, 24).

Significant child behavior problems are associated with insensitive parenting (25, 26) through a downward spiral in which both parents and children behave aversively (27). Problematic parent behaviors include harsh parenting, physical punishment, lax supervision and inconsistent discipline (24, 28).

Behaviorally based parenting programs reduce child behavior problems and increase positive parenting skills both short (29–33) and longer term (34, 35). Many studies also report significant improvements in maternal depression (36). However program content is varied and, despite their impact on child internalizing and externalizing behavior problems, effect sizes often remain small (37). It is clear from the development strong criteria for assessing the content, process and access to programs (the Blueprints for Violence Prevention criteria) that many programs achieve limited results due to lack of fidelity.

## 4. Attachment and behavior problems

Untreated early attachment difficulties are linked to subsequent hostility, hyperactivity, aggression, oppositional defiant disorder (38) and longer-term mental health problems and delinquency (39–42). Insecure attachment at age one predicts child aggression at age seven, and maternal criticism towards children at age four predicts more severe child aggression (43). Over 80% of pre-school children with behavior problems are insecurely attached to their parents (44) and teachers rate children with attachment problems as also having behavior problems poor school attendance and academic underachievement (45, 46).

The association between attachment and behavior problems is particularly relevant for looked after children (47) who have often been maltreated (48), have, by definition, had disrupted attachments and have three times the general rate of conduct disorders (49) with problems frequently established before entering care (50, 51). These problems present significant risk of placement breakdown (52) and are the major challenge for fostering services (53, 54).

Maternal depression predicts attachment and behavior difficulties (55) with 50%, of mothers of children with significant behavioral problems clinically depressed (33, 36, 56). Depressed mothers have lower rates of praise, fail to monitor children and spend less time playing with them (57–59). They have insensitivity to the cues of others (60, 61), poor observation skills (62, 63) and poor problem-solving (64, 65). They focus on deviant behavior and cannot describe behavior in detail (66–68) differentiating between parents of referred and non-referred children with behavioral challenges (69) and describing many aspects of parenting attachment difficulties.

Attachment relationship problems generally precede the emergence of behavior problems. However there are a number of possible explanations for the emergence of both problems: (1) Behavior problems can arise from parental insensitivity to children's cues causing children to escalate attention seeking problematic behavior; (2) Lack of positive attention can prompt children to seek alternative tangible, reinforcers such as foods or risky high adrenaline activities; (3) Some early child features, prematurity, disability, sleep/feeding difficulties, persistent crying, increase the risk of attachment problems because parents' caring behavior is not reinforced, increasing risks for child protection issues and behavior problems (70, 71); (4) Attachment and behavior problems may have independent causes, e.g., socioeconomic disadvantage (SED). However, SED is mediated through parenting behavior (27), impacting on children when parenting is compromised rather than independently causal. Furthermore the association between crime and structural factors is mediated by parental discipline, supervision, and attachment (72), so largely impacted by intervening family processes (73).

Hill et al. (74) identify two necessary parenting behaviors, discipline and safety and attachment security, associated with positive child outcomes. The effective response to challenging behaviors may be to ignore or to restrain a child, even if this increases the child's distress. For an attachment need, the response would be to offer comfort.

“*if family members interpret the attachment related distress of a child as a disciplinary issue, they may respond in a way that increases the distress. In the same way, the introduction of attachment concerns into a disciplinary process may lead to ineffective responses*” (74).

This is supported by Juffer et al. (75) whose meta-analysis targeting sensitive parenting found that attachment and discipline were both associated with fewer child behavior problems.

## 5. A behavioral analysis of attachment

Parental sensitivity can be understood in terms of principles of reinforcement. The “Learning Theory of Attachment” (76) describes how classical and operant conditioning processes explain attachment behavior. It assumes that the attachment behavior system in babies is innate, initially occurring during distress and the adult caring response reduces the stress. Bosmans et al. (76) describe ways in which physiological processes are triggered by caring adult responses:

*First, research shows that support decreases cortisol levels (UCRneg_decrease; e.g. [77]) which translates into a sense of relief (78). Second, support is supposed to increase oxytocin (UCRpos; e.g. [79]) translating into a sense of felt security and comfort (80). This has been described as a secure attachment state e.g. [81]) or as state trust (82, 83). Finally, oxytocin in turn activates the reward system and the secretion of dopamine (UCRpos) which motivates future affiliative behavior (84) and which plays a role in the conditioning process itself (85)* (76).

Infants support seeking behavior comes under the control of reinforcement through parents/caregivers removing discomfort and providing comfort through gentle touch, etc. These sensitive parental responses establish parent-child relationships (86, 87). The best predictors of secure infant attachment were explicit (contingent) behaviors that function as reinforcement, rather than implicit (non-contingent) parenting behaviors and interventions targeting them were more effective in changing caregiver sensitivity (88).

Further evidence for the ‘Learning Theory of Attachment’ (89) comes from experimental trials (80) and clinical work showing the development of secure attachment in children adopted after severe deprivation (90) or the disruption of secure attachments following severe life events (91).

## 6. Intervention

The frequent co-occurrence of behavior and attachment problems requires interventions that reduce both. In the past some attachment therapists/theorists did not accept the contribution of learning theory (7) or argued that behavioral challenges stemmed from underlying attachment causes and that behavioral parenting programs addressed symptoms not causes (92). Furthermore, despite strong evidence that behavioral programs reduce behavioral problems, some argued that non-violent discipline strategies such as time out, were inappropriate (54). Others suggest that behavioral programs worked with less damaged populations but were ineffective in addressing severe attachment difficulties (93).

The effectiveness of attachment-based interventions, in addressing behavioral, or co-occurring attachment and behavioral problems, is unclear (94), However, a Bakermans-Kranenburg et al. (95) meta-analysis of 88 sensitivity and attachment interventions, showed that “*interventions with a clear focus and a modest number of sessions are preferable…. Interventions with an exclusively behavioral focus on maternal sensitivity appear to be most effective not only in enhancing maternal sensitivity but also in promoting children's attachment security”* (pp. 212).

Establishing the parent as a positive reinforcer is an “essential part” of interventions for parents of children with significant behavior and attachment problems, and behavioral programs that include relationship building are more effective (96). This is particularly the case for interventions targeting children with severe behavioral problems (97) where the parent-child relationship has broken down. The most effective, strongly evidence-based, parenting programs for child behavior problems have Blueprint for Violence Prevention status (98), include Helping the Non-compliant Child (99), Parent Child Interaction Therapy (100) and the Incredible Years (101). All are based on the Hanf (102) two-stage model to address severe child behavioral challenges. Previous psychodynamic therapy did not involve parents and lacked evidence (103).

Hanf started with unconditional relationship building conceptualized as establishing/re-establishing the parent as a reinforcer by association with activities that the child found reinforcing. By pairing a behavior that is reinforcing (play) with a less frequently occurring behavior (positive parental attention) the latter acquires reinforcing properties (104). For example, children learn to like eating olives or other novel experiences through exposure, in the presence of someone who is reinforcing to them and reinforces their engagement in these activities.

Hanf strengthened parent-child relationships before introducing discipline strategies, subsequently enabling the parent to reinforce appropriate behavior and set limits that the child accepted. Hanf had specific criteria for rates of attending and praising child behavior before moving to the second stage. Once established this child-led component continued throughout the intervention. The second stage introduced contingent praise and rewards for desirable behavior, consistency, positive instruction giving, setting clear limits, extinction of undesirable behaviors through ignoring and, where necessary, time out and consequences.

Hanf directly coached and reinforced parents, modelling and prompting desired parenting behavior. This is also how the most effective behaviorally based parenting programs are delivered (105) with parents learning skills through discussion, realistic goal setting and actual or role-play practice.

Many randomized controlled trials (RCTs) and systematic reviews present quantitative & qualitative evidence for Hanf derived programs (29) especially for children aged 2–9 years (96). However, relatively little evidence reports their impact on attachment relationships. Even a recent study comparing behavioral and attachment-based program impact on children's externalizing problems used no attachment-based measures (106).

Three studies have reported impact of Hanf derived programs on both child behavior and attachment related behaviors. O'Connor et al. (83) analyzed observational data involving three parent-child interaction tasks, from an RCT of the Incredible Years parenting intervention (107) to test whether it independently, promoted attachment-based parenting. Many studies collapse observational ratings across different tasks (108). However, O'Connor et al. (83) coded three tasks, free play, structured challenge and tidy-up, separately for attachment and parenting behaviors. Two attachment measures, sensitive responding and mutuality, were coded (109). Parental behavior management skills were coded using the Parent Behavior Coding Scheme (110, 111). Changes in child-centered parenting were robust and partly distinct from improvements in attachment-based sensitive responding and varied across tasks.

“*there was not strong evidence of coordinated change between the child-centered and Sensitive Responding parenting measures:(a) correlation between change scores was modest; (b) there was not significant evidence of mediation for Sensitive Responding; and (c) whereas changes in child-centered behaviors were most evident in the free play and LEGO challenge interaction tasks, changes in Sensitive Responding were strongest in the tidy-up task. That implies that the social learning theory–based intervention produced additional and not merely overlapping changes in attachment-related caregiving*” (83).

A trial of the Infant Behavior Program (IBP) (112), a version of Parent–Child Interaction Therapy (100) evaluated attachment-based caregiving behaviors by coding videotaped infant-led play (113). Behaviorally based parenting skills correlated moderately with attachment-based caregiving behaviors, with direct effects on warmth and sensitivity, mediated by post-intervention increases in parenting skills. They concluded that the behavioral skills had a broader impact on attachment-based caregiving.

Further evidence of impact on attachment relationships comes from an RCT by Fisher and Kim (114) of the Multidimensional Treatment Foster Care Program for Preschoolers (MTFC-P) (115) a pre-school version of Multidimensional Treatment Fostercare Program (MDTF) (116). They demonstrated significant positive impact on the attachment-related behavior of maltreated fostered pre-school children toward caregivers over a 12-month period following a new placement.

Meta-analyses support the importance of relationship building in behavioral parenting programs with relationship building and behavior management each associated with stronger program effects (117). However, because these components are often integrated (96) it was difficult to interpret findings when the presence (versus absence) of the other component was not taken into account. However, a meta-analysis of 156 behavioral parenting RCTs (97) tested the effects of programs that integrated relationship building and behavior management versus those that only taught behavior management. The programs included universal, preventive, targeted and treatment populations. Combined behavioral management and relationship building was not superior overall but was superior in treatment trials and inferior in preventive trials. Parents accessing behavior management advice in universal non-clinical settings addressing normal behavioral challenges generally do not have the compromised attachment relationships typical of the coercive relationships between parents and children with clinical levels of problems (26). This makes sense since it is in settings, where children have significant behavioral problems, that attachment relationships are most likely to be compromised and need addressing.

Using the same RCTs, Leijten et al. (118) coded each study for 26 intervention techniques. Three core techniques showed stronger effects overall in reducing disruptive behavior, positive reinforcement, praise as a specific operationalization of positive reinforcement, and natural/logical consequences. However, they were all associated with stronger effects in treatment samples. Similar effects to those from the earlier meta-analysis (97) were found for four additional techniques: relationship building as a general technique, parent-child play as operationalization of relationship building, active listening, and parental self-management, which would contribute to enabling parents to meaningfully reinforce other behaviors. All four showed stronger effects in treatment samples and weaker or no effects in prevention trials (118).

## 7. Conclusion

Attachment relationships for young children are significantly affected by the parenting that they experience and the impacted brain cortisol patterns can become harder to change over time (1). Attachment based interventions have often failed to focus on, or measure, their impact on the behavioral challenges and behaviorally based interventions, that have been shown to impact attachment relationships, have so far failed to explore changes in the underlying brain function and cortisol levels that are impacted by poor early parenting.

The formation of attachment can be understood as a behavioral process (76) that, for children with significant behavioral problems requires relationship building to be addressed as part of the program. This can independently improve attachment relationships and reduce behavioral problems. The contribution of behavioral interventions in addressing both behavioral and attachment challenges is relatively under-researched and could benefit the many children with either/both difficulties.

This paper reports on the frequent co-occurrence of child attachment and behavioral problems and presents a “Learning Theory of Attachment” (76) and theoretical reasons why Hanf derived programs, that include relationship building reduce both attachment and behavioral problems. Until recently, there was limited evidence to demonstrate this, however, Blizzard et al. (113) and O'Connor et al. (83) both showed improvements in attachment related parenting behaviors that were partly independent of behavioral parenting skills and Fisher and Kim (114) showed the impact of a behaviorally based program on the attachment relationships of looked after children. The two Leijten et al. papers (97, 118), showed that relationship building components added significantly to intervention outcomes for child behavior problem treatment samples. The strongest evidence-based behavioral interventions, that include behaviorally based relationship building, alongside other learning theory principles, stem from the work of Hanf (96, 103). Future research must explore the unique contribution that behaviorally based programs make to improving attachment relationships, including their impact on brain function in relation to regulatory processes, alongside increasing parental use of behavioral parenting techniques and reducing child behavioral problems.

## Data Availability

The original contributions presented in the study are included in the article/Supplementary Material, further inquiries can be directed to the corresponding author.
