# Correction: Attachment, behavior problems and interventions

**DOI:** 10.3389/frcha.2025.1650383

**Published:** 2025-07-24

**Authors:** Judy Hutchings, Margiad E. Williams, Patty Leijten

**Affiliations:** ^1^ Centre for Evidence Based Early Intervention (CEBEI), School of Human and Behavioural Sciences, Bangor University, Bangor, United Kingdom; ^2^ Research Institute of Child Development and Education, University of Amsterdam, Amsterdam, Netherlands

**Keywords:** childhood, attachment, behavior problems, parenting programs, behavioral analysis

In the published article References (86–94) were not cited in the article. The citation has now been inserted in 5. A behavioral analysis of attachment paragraphs 3 and 4 and should read:

“Infants support seeking behavior comes under the control of reinforcement through parents/caregivers removing discomfort and providing comfort through gentle touch, etc. These sensitive parental responses establish parent-child relationships (86, 87).

The best predictors of secure infant attachment were explicit (contingent) behaviors that function as reinforcement, rather than implicit (non-contingent) parenting behaviors and interventions targeting them were more effective in changing caregiver sensitivity (88).

Further evidence for the ‘Learning Theory of Attachment’ (89) comes from experimental trials (80) and clinical work showing the development of secure attachment in children adopted after severe deprivation (90) or the disruption of secure attachments following severe life events (91)” and section 6. Intervention paragraphs 1 and 2 and should read:

“The frequent co-occurrence of behavior and attachment problems requires interventions that reduce both. In the past some attachment therapists/theorists did not accept the contribution of learning theory (7) or argued that behavioral challenges stemmed from underlying attachment causes and that behavioral parenting programs addressed symptoms not causes (92). Furthermore, despite strong evidence that behavioral programs reduce behavioral problems, some argued that non-violent discipline strategies such as time out, were inappropriate (54). Others suggest that behavioral programs worked with less damaged populations but were ineffective in addressing severe attachment difficulties (93).

The effectiveness of attachment-based interventions, in addressing behavioral, or co-occurring attachment and behavioral problems, is unclear (94)”.

The remainder of the reference citations are correct and should read:

86. Gewirtz JL. Identification, attachment, and their developmental sequencing in a conditioning frame. In: Gewirtz JL, Kurtines WM, editors. Intersections with Attachment. Hillsdale, NJ: Erlbaum (1991). p. 247–55.

87. Gewirtz JL, Peláez Nogueras MBF. Skinner's legacy to human infant behavior and development. Am Psychol. (1992) 47:1411–22. doi: 10.1037/0003-066X.47.11.1411

88. Dunst CJ, Kassow DZ. Caregiver sensitivity, contingent social responsiveness, and secure infant attachment. J Early Intensive Behav Interv. (2008) 5:40–56. doi: 10.1037/h0100409

89. Bosmans G, Waters TEA, De Winter S, Hermans D. Trust development as an expectancy-learning process: testing contingency effects. PLoS One. (2019) 14:e0225934. doi: 10.1371/journal.pone.0225934

90. Van IJzendoorn MH, Juffer F. The Emanuel Miller memorial lecture 2006: adoption as intervention. Meta-analytic evidence for massive catch-up and plasticity in physical, socio-emotional, and cognitive development. J Child Psychol Psychiatry. (2006) 47:1228–45. doi: 10.1111/j.1469-7610.2006.01675.x

91. Sroufe LA, Egeland B, Carlson EA, Collins WA. The Development of the Person: The Minnesota Study of Risk and Adaptation from Birth to Adulthood. New York, NY: Guilford Publications (2005).

92. O’Connor TG, Matias C, Futh A, Tantam G, Scott S. Social learning theory parenting intervention promotes attachment-based caregiving in young children: randomized clinical trial. J Clin Child Adolesc. (2013) 42:358–70. doi: 10.1080/15374416.2012.723262

93. Green J, Goldwyn R. Annotation: attachment disorganisation and psychopathology: new findings in attachment research and their potential implications for developmental psychopathology in childhood. J Child Psychol Psychiatry. (2002) 43:835–46. doi: 10.1111/1469-7610.00102

94. Axford N, Bjornstad G, Matthews J, Heilmann S, Raja A, Ukoumunne OC, et al. The effectiveness of a therapeutic parenting program for children aged 6–11 years with behavioral or emotional difficulties: results from a randomized controlled trial. Child Youth Serv Rev. (2020) 117:105245. doi: 10.1016/j.childyouth.2020.105245

The authors apologize for this error and state that this does not change the scientific conclusions of the article in any way. The original article has been updated.

